# Prevalence and Potential Risk Factors for the Acquisition of Antibiotic-Resistant *Staphylococcus* spp. Bacteria Among Pastoralist Farmers in Kajiado Central Subcounty, Kenya

**DOI:** 10.1155/2023/3573056

**Published:** 2023-04-11

**Authors:** Edidah Ong'era, John Kagira, Naomi Maina, Daniel Kiboi, Kenneth Waititu, Lynda Michira, Maina Ngotho

**Affiliations:** ^1^Department of Biochemistry, Jomo Kenyatta University of Agriculture and Technology, Nairobi, Kenya P.O. Box 62000-00200; ^2^Department of Animal Sciences, Jomo Kenyatta University of Agriculture and Technology, Nairobi, Kenya P.O. Box 62000-00200; ^3^Department of Animal Science, Institute of Primate Research, P.O. Box 24481 Karen 00502 Nairobi, Kenya; ^4^Department of Clinical Studies, University of Nairobi, Nairobi, Kenya P.O. Box 30197-GPO

## Abstract

Antimicrobial resistance (AMR) is a growing health problem globally. To address this challenge, there is a need to generate baseline data on the prevalence and AMR profile of the main disease-causing bacteria. Here, we interrogated the prevalence of bacteria in the nasal cavity of healthy pastoralists in Kajiado Central Subcounty, Kenya, and the occurrence of AMR in Staphylococcus isolates among the study subjects. Nasal swabs from 176 pastoralists were cultured, and the bacteria isolates identified using standard phenotypic and biochemical bacteriological methods. Among the obtained 195 isolates, the most prevalent isolates were coagulase-negative Staphylococcus (CoNS) (44.9%), followed by *Enterococci* spp. (43.2%) while *Staphylococcus aureus* prevalence was 8%. Antimicrobial sensitivity of the *Staphylococcus* spp. isolates to 14 antibiotics representing six antibiotic groups was undertaken using the Kirby-Bauer disk diffusion method. Among the CoNS, the highest resistance was reported in amoxicillin (78.7%) and ceftazidime (76%), while the most resistance for *S. aureus* was reported in ceftazidime (100%), amoxicillin (71.4%), and streptomycin (71.4%). From an administered questionnaire looking at gender, animal contact frequency, history of hospital visitation and antibiotic usage, and habitual intake of raw milk, the study showed that male participants had a higher risk of carrying multiple drug resistant (MDR) bacteria than females (*p* = 0.02, OR = 1.3). Likewise, habitual intake of raw milk was significantly associated MDR acquisition (*p* = 0.02, OR = 1.82). This study reveals a high prevalence of AMR Staphylococcus isolates in the study area laying a foundation for further analysis of molecular characterization of the observed resistance as well as the development of interventions that can reduce the occurrence of AMR in the study area.

## 1. Introduction

The upper respiratory tract harbors commensal and potentially pathogenic bacteria, i.e., *Staphylococcus* spp., *Moraxella* spp., *Corynebacterium* spp., *Streptococcus* spp., and *Haemophilus* spp. [1]. The commensal bacteria function as competitors to pathogenic bacteria and continuously prime the immune system against the pathogenic bacteria [2]. The *Staphylococcus* spp. colonizing the nasal cavity are divided into coagulase-positive (*S. aureus*) and coagulase-negative (CoNS) and account for a variety of infections and diseases in both humans and livestock [3]. In African countries, Staphylococcal carriage ranges from 8.0 to 57% in both patients and healthy populations [4–6].

The first-line treatment for Staphylococcal infection is penicillinase-stable penicillin [7, 8]. However, resistance to this class of antibiotics was first identified in1960s in *Staphylococcus aureus* [9]. AMR in Staphylococcus is highest in low-income countries and low middle-income countries [10–12]. Currently, methicillin-resistant Staphylococcus (MRS) pathogens are among the leading causes of death with a prevalence of 1-80% worldwide [13, 14]. MRS is majorly caused by the acquisition of the staphylococcal cassette chromosome *mec* (*SCCmec*) comprised mainly of *mecA* gene or its homologues (*mecB* or *mecC*) which encodes for a novel penicillin-binding protein (PBP2a) with a lower affinity to the beta-lactam ring and the site-specific recombinase gene (ccrA/B/C). After its first recovery in the 1980s, the diversity of the SCCmec was noted first in the 1990s, and afterward, more diversity was detected between 2001 and 2008 [15]. To date, fourteen types of *SCCmec* (I-XIV) have been reported [16]. The first community-associated (CA-MRS) was detected in the 1980s [17]. There is a steady increase of CA-MRS in the 1990s and early 2000s [7, 15, 18–20]. CA-MRSA has majorly been associated with *SCCmec* types IV, V, and VI [16, 21–23].

The integrons have been reported to foster the spread of AMR in *Staphylococcus* spp. given their ability to naturally clone and express resistant genes. Integrons contain up to 40 genes associated with resistance to beta-lactams, sulfonamides, aminoglycosides, chloramphenicol, and macrolides [24, 25]. The occurrence of integrons in HA-MRS isolates was first reported in 2007 in South China [26]. Since then, integrons have been identified in antibiotic-resistant staphylococcal isolates largely in the Asian continent [27–31]. However, data on integron associated Staphylococcus AMR in other parts of the world especially in the developed countries and Africa are lacking. Coexistence of class 1/2 integron gene cassettes and *SCCmec* in MRS has been reported occasionally [28, 32–34]. The simultaneous existence of both mechanisms is anticipated to increase the adaptability of microbes to antibiotic selective pressure speeding up the spread and survival fitness of resistant Staphylococcus while fostering MDR evolution [32–35].

MRS infections are treated using a combination of penicillin and vancomycin [36, 37]. However, suboptimal responses to vancomycin treatment, vancomycin intermediate susceptible Staphylococcus (VIS) minimum inhibitory concentration (MIC 4-8 *μ*g/ml), were first detected in Japan in 1997 whereas total resistance to vancomycin, vancomycin-resistant Staphylococcus (VRSA) MIC ≥ 16 *μ*g/ml, was detected in the USA in 2002 [38, 39], whereas the VIS is thought to be because, by cell wall thickening, VRS is caused by the acquisition of the Van operon from *Enterococcus faecalis* bacteria [40, 41]. Since their emergence, VIS and VRS isolates have been detected in different parts of the world albeit at low percentages [42–45]. Penicillin-resistant Staphylococcus on the other hand is caused by a *blaz* gene carried in the R plasmids which encodes for the penicillinase enzyme that hydrolyses the *β*-lactam ring [46, 47]. On the other hand, accumulation of mutations on the drug target genes has been identified as a main cause of resistance for protein and DNA replication inhibitors [48, 49].

The emergence of antimicrobial resistance is mainly caused by the indiscriminate use of antibiotics for treatment/prophylaxis in both human and animal ecologies [50]. Globalization and lifestyle factors such as the intake of raw/undercooked animal products, low hygiene level, and high population density as well foster the spread of antimicrobial resistance [5, 51]. In Africa, AMR in *Staphylococcus* spp. ranges from 12 to 80% [52–54]. In Kenya, *Staphylococcus* spp. AMR ranges between 20 and 84.1% [54–57] with a steady increase from <50% in the 2000s to >50% by 2020 in Staphylococcus antimicrobial resistance [55, 57–63], whereas most *Staphylococcus* spp. AMR studies in Kenya have been conducted in the urban hospital setting (Nairobi, Kiambu, Kilifi, Kisumu, and Kericho); majority of the studies [60, 64, 65] investigating antibiotic resistance in rural areas, especially among pastoralist communities such as the Maasai, who use large quantities of antibiotics for veterinary care with >95% being self-administered and up to 75% carried out without professional consultation, are lacking or poorly documented [51]. The current study is aimed at determining the prevalence, risk factors, and detection of *mecA* or *mecC* antibiotic-resistant marker in Staphylococcus isolates obtained from the anterior nares of livestock farmers in Kajiado Central Subcounty in Kenya.

## 2. Material and Methods

### 2.1. Study Area

The study was undertaken in the Kajiado Central Subcounty region in Kajiado County, Kenya, located in the southern part of Kenya, approximately 80 km from Nairobi City and borders Northern Tanzania: 1.83874 latitudes and 36.79135 longitudes. The subcounty is divided into five wards: Matapato South, Matapato North, Purko, Ilidamati, and Dalalekutuk, which were included in this study. Kajiado Central experiences bimodal rainfall ranging from 500 to 1200 mm, with short rains occurring from October to December and long seasons between March and May, and an average temperature of 25°C. The total human population of the subcounty is 161,862, most of which are of the Maasai community and are pastoralists. The total livestock population in the subcounty as per the 2019 census was 802,898 [66].

### 2.2. Study Design

A descriptive cross-sectional study design was used. The study sample size was determined using the Thrusfield method [67], which showed that a minimum of 150 participants were sufficient for the current study. A total of 176 participants were sampled. The sampling unit of interest was household members, which were selected randomly from a sampling frame provided by the county extension and public health officers. Based on the human population in specific wards, the study participants were from Matapato South (*n* = 54), Matapato North (*n* = 51), Purko (*n* = 25), Ilidamati (*n* = 30), and Dalalekutuk (*n* = 16) wards (Table 1).

### 2.3. Nasal Swab Collection and Administration of the Questionnaire

Nasal swabs were collected, as described previously [68]. Briefly, the swabs were collected using a sterile nasal swab (BD BBL™ CultureSwab™) dipped in normal saline by slightly swabbing both anterior nares of the participants and transferred to the transport media (Scharlab S.L, Spain). The sample was labelled and transported back to the microbiology laboratory based at the Institute of Primate Research, Kenya, in a cool box. The data on the risk factors, age, gender, history of antibiotic use, hospital visitation < 3 months prior to the study, intake of raw milk, and animal contact, were obtained through a structured questionnaire administered in local Maasai language to the study participants by the investigator.

### 2.4. Culture and Identification of Bacteria

To identify the bacteria in the collected samples, the nasal swabs were first inoculated on MacConkey agar (Scharlab S.L, Spain) and sheep blood agar (Scharlab S.L, Spain) and incubated at 37°C for 18-24 hrs. The inoculated sheep blood agar plates were incubated in an environment enriched with 5% CO_2_. Colonies were then identified based on morphological appearance on the respective agar plates, Gram stain reaction, and biochemical tests [69]. The catalase test was used to distinguish staphylococci from streptococci. Catalase-positive staphylococci were further subjected to a coagulase test to distinguish *S. aureus* from coagulase-negative Staphylococcus. Pure isolates of *S. aureus* and coagulase-negative Staphylococcus were then subjected to antimicrobial susceptibility testing [69].

### 2.5. Antibiotic Susceptibility Testing

To determine the antibiotic susceptibility profiles of the isolated *S. aureus* and coagulase-negative Staphylococcus, a standardized Kirby-Bauer disk diffusion method utilizing the Mueller-Hinton agar (Scharlab S.L, Spain) plate technique was performed as per the Clinical and Laboratory Standards Institute guidelines [70]. Briefly, a uniform suspension of each isolate was prepared and adjusted to a concentration equivalent to 0.5 McFarland with sterile normal saline. Each suspension was evenly spread on two Mueller-Hinton agar (Scharlab S.L, Spain) plates followed by placing seven antibiotics disks on every plate. A total of fourteen antibiotic disks representing six antibiotic groups, beta-lactams (amoxicillin 30 *μ*g, oxacillin 1 *μ*g, Augmentin 20/10 *μ*g, ceftriaxone 30 *μ*g, ceftazidime 30 *μ*g, cefoxitin 30 *μ*g, and cefotaxime 30 *μ*g), tetracyclines (tetracycline 30 *μ*g), glycopeptides (vancomycin 30 *μ*g), aminoglycopeptides (gentamicin 10 *μ*g and streptomycin 10 *μ*g), macrolides (erythromycin 15 *μ*g and clindamycin 2 *μ*g), and fluoroquinolones (ciprofloxacin 30 *μ*g), were placed on the plates as per the CLSI guidelines. Measurements of the inhibitory zones were done, and the results were interpreted according to the CLSI guidelines. The results were reported as resistant, intermediate, or susceptible to specific antibiotics [70].

### 2.6. Detection of the *mecA* and *mecC* Genes Using Singleplex PCR

DNA from the resistant bacteria was extracted using the heat lysis method. Briefly, one bacterial colony was suspended in 500 *μ*l of nuclease-free water, heated at 95°C for 10 minutes followed by freezing at -80°C for 5 minutes. The DNA was harvested by centrifugation at 1200 rpm for 10 min, the supernatant was picked, and pellets were discarded. The extracted DNA was subjected to PCR for detection of *mecA* and *mecC* genes using primers *mecA* 513 bp C05/C06 (F-AAA ATC GAT GGT AAA GGT TGG C) and D09/D10 (R-AGT TCT GCA GTA CCG GAT TTG C) and *mecC* 718 bp (GAAAAAAAGGCTTAGAACGCCTC CCTGAATCTGCTAATAATATTTC). A PCR protocol of 25 *μ*l final volume containing 2x Taq polymerase master mix (NEBNext, New England Biolabs, Inc.) was adopted to run 94°C for 5 min followed by 35 cycles of 94°C for 30 seconds, 58°C for 40 seconds, 72°C for 40 seconds, and a final extension at 72°C for 5 minutes. The products of PCR amplification were analyzed using 2% agarose gel electrophoresis 1x tris acetate ethylene (TAE) diamine tetra acetic acid buffer stained with SYBR Sate TM fluorescent dye. Positive Staphylococcus reference strains *S. aureus ATCC* 25923 and *S. epidermidis ATCC* 12228 were used as controls. A template control was added as a negative control in both assays.

### 2.7. Ethical Approval

The ethical clearance was granted by the JKUAT Ethics Review Committee (JKU/2/4/896B) and Kajiado County Medical and Public Health Service Department. The livestock farmers were informed about the study, and written consent was subsequently obtained from those more than 18 years while assent was sort from the parent/guardian for the minors.

### 2.8. Data Analysis

The obtained data was entered and analyzed using Microsoft Excel. The proportion of the identified bacteria species was determined by dividing the total number of the specific bacteria by the sample size. Analysis of the odds ratio (OR) and *p* value was done to evaluate the association between risk factors (age, history of hospital visitation, contact with cattle, history of antibiotic usage, intake of raw milk, and gender factors) and presence of MDR Staphylococcus bacteria for a potential link to acquisition. A *p* value of < 0.05 was considered statistically significant.

## 3. Results

### 3.1. Characteristics of Study Participants

A total of 176 study participants were sampled from the study area. Majority of the respondents were males (61.4%) with 82.4% of them reporting frequent contact with livestock. Of the study participants, only 26.7% recorded having visited a hospital within 3 months prior to this study, while 22.7% of the participants consumed raw milk habitually with the majority (40%) being <20 years. Other investigated predisposing factors are outlined in Table 2.

### 3.2. Prevalence of Nasal Bacteria

A total of 195 bacterial isolates were obtained from the collected 176 nasal swabs. All (100%) participants' nares were colonized by various bacteria. The most common isolates were coagulase-negative Staphylococcus (CoNS) (44.9%) and *Enterococci* spp. (43.2%) (Table 3). The *Staphylococcus aureus* prevalence was 8%. Other isolates obtained from the study are outlined in Table 3. Respondents from Matapato South and Matapato North had the highest (50%) prevalence of CoNS, while *Enterococcus* spp. were more common in respondents from Matapato South ward.

### 3.3. Antibiotic Resistance

The obtained *Staphylococcus* spp. were further investigated for antimicrobial susceptibility. Among the CoNS, the highest resistance was amoxicillin (78.7%), followed by ceftazidime (76%) and oxacillin (49.3%). Majority of the CoNS isolates, however, were sensitive to Augmentin (88.0%) and ciprofloxacin (84%). Resistance to other tested antibiotics is shown in Table 4.

For *S. aureus*, all (100%) isolates were resistant to ceftazidime. The percentage of *S. aureus* resistant to amoxicillin (71.4%), streptomycin (71.4%), and vancomycin (64.3%) was also high. The *S. aureus* isolates resistant to oxacillin (28.6%) and cefotaxime (21.4%) resistance were lower compared to the CoNS isolates. In contrast, *S. aureus* isolates were highly (100%) susceptible to Augmentin. The summary of the sensitivities of *S. aureus* to individual antibiotics is shown in Table 4.

From the study findings, majority of the CoNS isolates were resistant to amoxicillin in combination with oxacillin 61/79 (77.2%) and ceftazidime 56/79 (70.9%) while *S. aureus* major resistance combination was observed in the amoxicillin and ceftazidime 10/14 (71.4%), streptomycin (42.9%), ceftazidime in combination with streptomycin 7/14 (50.0%), and vancomycin 5/14 (35.7%). Other major observed resistance combination is shown in Table 5.

Occurrence of MDR was observed across both species of Staphylococcus. In this study, most of the MDR involved beta-lactams and cephalosporins. For CoNS, the MDR involved beta-lactams and other classes of antibiotics: cephalosporins (57/79, 72.2% isolates), macrolides (37/79, 46.8% isolates), oxytetracycline (27/79, 34.2% isolates), aminoglycosides (16/79, 20.3%), and glycosides (15/79, 19%). For the *S. aureus*, MDR was observed majorly in beta-lactams and cephalosporins (13/14, 92.9%), aminoglycosides (7/14, 50% isolates), oxytetracycline (2/14, 14.29% isolates), and glycosides (3/14, 21.4% isolates). Resistance for the beta-lactam class was consistently high across all wards.

The prevalence of resistant *Staphylococcus* spp. strains varied across the study wards. Study participants from Matapato South ward had bacteria resistant to all classes of antibiotics. Details of the distribution of resistance to the six classes of antibiotics tested are shown in Table 6.

### 3.4. Relationship between Risk Factors and Prevalence of Multidrug Resistance in Bacteria Isolated from the Study Participants

The antibiotic susceptibility results were used to evaluate the potential association between various factors: gender, age, consumption of raw milk, animal contact, antibiotic usage, hospital visits, and the occurrence of MDR bacteria in nares (Table 7. From the analysis, male participants had a higher risk of carrying MDR bacteria than females (*p* = 0.02, OR = 1.3), while habitual intake of raw milk was significantly associated with a higher prevalence of MDR bacteria (*p* = 0.02, OR = 1.82). Majority of the study participants (82.4%) were frequently in contact with livestock; however, this factor was not associated with the occurrence of drug resistance bacteria (*p* = 1, OR = 9.7). The relationship between the prevalence of various risk factors and the occurrence of MDR bacteria is outlined in Table 7.

### 3.5. Prevalence of *mecA* and *mecC* Genes among Methicillin-Resistant Staphylococcus Isolates

The phenotypically methicillin-resistant isolates were further investigated for the genotypic presence of *mecA* and *mecC* genes. The study showed that 16% of the CoNS isolates were mecA positive. Eight percent (8%) of the CoNS isolates harbored the *mecC* while the *S. aureus* isolates lacked both the *mecA* and *mecC* genes.

## 4. Discussion

The current study was geared at determining the spectrum of bacteria colonizing the nasal cavity of a pastoralist community in Kajiado Central Subcounty in Kenya as well as determine the antibiotic sensitivity of the obtained Staphylococcus isolates. First, CoNS, *Enterococci spp.*, and *S. aureus* were the dominant bacteria in the nasal cavity of the study subjects with CoNS contributing close to half of the isolates. The dominance of these bacteria is not unique to these settings. Different studies across the world (Brazil, Saudi Arabia, Kuwait, Middle East, and Egypt) have reported similar trends [71–73]. The dominance of these bacteria is largely due to their commensal nature in humans, mainly on the skin, nasal cavity, and gut [2, 74]. The prevalence of *S. aureus* is however lower compared to that recorded elsewhere in hospital settings and among healthcare workers in Kenya [4, 56] and across communities in East Africa and sub-Saharan regions in Africa [55, 75, 76]. The difference may have been influenced by the difference in study, laboratory, experimental setting, and climate factors where the high temperature in the study area could have reduced *S. aureus* colonization [77, 78]. Moreover, the high rates of CoNS isolated in this study might have lowered *S. aureus* colonization by secreting molecules that negatively affect the virulence of *S. aureus* [79, 80].

Secondly, we show that most of the circulating Staphylococcus isolates in the study population are resistant to at least one antibiotic tested in our study. The highest resistance was recorded in the penicillin which is in line with other studies across the world [54, 56, 60, 81–85]. High penicillin resistance is linked with overuse of these antibiotics in treatment of bacterial infections especially in livestock [84–86]. In our opinion, HGT and interspecies transfer within the livestock and human ecology might have caused the observed resistance in the human population. Methicillin resistance among the CONS isolates was close to that reported in Nigeria (65%) [87] and Ghana (60-93%) [88, 89] but slightly lower than that recorded in some sub-Saharan countries (<90%) [90–92]. This resistance is however higher than that recorded in hospital settings in some parts of Kenya (21-30%) [93, 94] suggesting a higher circulation of MRS isolates in the study population compared to the other places which is alarming given the weak association of the history of antibiotic usage and hospital visitations to emergence and spread of MDR in this community. However, methicillin resistance for *S. aureus* in our study is comparable with that recorded elsewhere in the country in hospital, livestock, and community settings (1-90%) [4, 60, 83, 95].

Interestingly, resistance to vancomycin in the study area was higher than that recorded elsewhere in the world: 0-11% in Kenyan hospital setting [54, 82, 96] and 0-16% in the rest of the world [53, 97, 98]. The resistance is however lower than that identified among camel slaughterhouse workers in Ethiopia (54%) [99]. Vancomycin resistance has been linked with presence of *vanA* operon especially in enteric bacteria. Molecular investigation of the causes of the observed vancomycin resistance will be beneficial especially in establishing possibility of HGT of vancomycin resistance gene to the *Staphylococcus* isolates given the codominance of the two bacteria in the study area. Importantly, the high vancomycin resistance in the current population compared to other studies might be partly due to low sample size especially among the *S. aureus* which limits the statistical power of the study. Of note, resistance of the Staphylococcus isolates across all six antibiotic classes tested (glycopeptide, aminoglycosides, macrolides, tetracycline, and quinolones) was noted which is alarming. Given the high susceptibility of the Staphylococcus isolates to Augmentin compared to the rest of the tested antibiotics, this drug can serve as an alternative drug for the treatment of staphylococcal infections in this population.

The presence of *mecA* and *mecC* genes in some of the CoNS isolates confirms the presence of *SCCmec*-associated MRS in the study area. The *mecA* gene is the gold standard for MRS; thus, occurrence of *mecA* gene and *mecC* gene in our study was expected. Although the *mecC* gene has been detected in various clinical studies across the world [100–103], this is the first study to report *mecC* gene resistance in Kenya. The presence of *mecA* and *mecC* genes in the methicillin-resistant isolates forms a basis for further molecular work especially *SCCmec* typing in the current study population. The absence of both the *mecA* and *mecC* genes in some of the MRS isolates is not unique to this setting; some studies have reported similar findings or lack of one of these genes in methicillin-resistant isolates [101, 104–106]. Thus, the possibility of a different MRS mechanism in the study area is highly probable which needs further investigation. Some studies that have explored further *mecA* and *mecC* negative isolates have identified the presence of the *mecB* homologue, hyperproduction of *β*-lactamase, and production of modified PBPs [17, 107–109]. Further, class 1 and 2 integrons containing *dfrA12-orfF-aadA*2, *dfrA17-aadA5*, *aadA2*, and *aadB*, *oxa2*, *aacA4*, *orfD-aacA4-catB8*, *aadB-catB3*, *orfD-aacA4* and *aadB-aadA1-cmlA6*, *dhfrA1-sat2-aadA1*, *dhfrA11*, *dhfrA1-sat2* gene cassettes, respectively, have been detected in resistant Staphylococcus isolates in the past [32–34]. Given the ability of the integrons to carry resistant genes for *β*-lactams, further investigation of the presence of these classes of integrons in the current study population will be necessary.

Multidrug resistance (MDR) was recorded in both CoNS and *S. aureus* majorly to the beta-lactams, macrolides, and aminoglycosides. Although most studies in Kenya and East Africa have focused on multiple drug resistance in livestock, food, and nosocomial infections, our findings are comparable to these studies [84, 110–112]. MDR resistance has been attributed accumulation of multiple drug resistant genes or overexpression of resistance genes encoding for efflux pumps and acquisition of less susceptible genes [113–117]. Investigation of the risk factors associated with MDR linked livelihood factors to the acquisition of MDR Staphylococcus isolates. Habitual intake of raw milk had a significant impact on the acquisition of resistant MDR Staphylococcus bacteria which is in accord with other studies conducted elsewhere in the world [118–120]. *Staphylococcus* spp. bacteria have been one of the foodborne pathogens occurring in raw milk; thus, the link between raw milk intake and occurrence of MDR in the study participants is not surprising. Also, from the study, the males had higher odds of acquiring resistant bacteria compared to female which is in line with other studies [121–123]. The likelihood of the males to acquire MDR bacteria may be linked to the occupational differences between the two genders in the Maasai community where the males are more involved in animal husbandry compared to the females in this community, as well as differences between hygiene levels in the two genders.

Although antibiotic usage in the current study was not significantly associated with the acquisition of antibiotic-resistant strain which differs with other studies [51, 124, 125], the study result relied heavily on study participant honesty which was dependent on their memory, a factor that might have limited our study findings. Most pastoralists are known to extensively use antibiotics in the management of livestock diseases with lack of withdrawal period during the medication period. This may have led to consumption of antimicrobial drugs eventually through milk and meat products. In such scenarios, HGT between livestock ecology and human is possible. The link of livelihood factors to acquisition of MDR acquisition necessitates proper understanding of the diverse beliefs in this community, and how they affect AMR acquisition and spread that will be essential in designing a proper AMR intervention to mitigate antibiotic resistance spread.

## 5. Conclusions

We outline three major findings from this study. First, most of the circulating nasal bacteria in the Kajiado Central were CoNS with some containing the *mecA* and/or *mecC* antibiotic-resistant markers, suggesting the possibility of factors promoting high nasal colonization of *Staphylococcal* spp. in humans that need further investigation. Second, we record a high prevalence of MDR for most of the antibiotics currently used for staphylococcal infection treatment. Lastly, livelihood factors (intake of raw milk and gender) were the major contributors to the acquisition of multidrug resistance strains, while antibiotic pressure and history of hospital visitation < 3 months prior to this study did not significantly impact the acquisition of resistant bacteria strains in the human population. These findings provide a basis to integrate social studies into antimicrobial resistance in this community to understand the cultural, social, and economic factors influencing the acquisition of antimicrobial resistance in this study area. The study also gives insight on the presence of *mecA*/*mecC* methicillin-resistant Staphylococcus strains laying a foundation for further investigation of molecular mechanisms in the observed resistance as well as building up on efforts to mitigate antimicrobial resistance at both the national and global scales. This will aid in the development of appropriate antimicrobial resistant mitigation strategies.

## Figures and Tables

**Table 1:** The number of study participants as distributed per ward in Kajiado Central Subcounty, Kenya.

Wards	Total respondents sampled	Number of homesteads sampled
Matapato North	54	12
Matapato South	51	8
Purko	25	5
Ilidamati	30	4
Dalalekutuk	16	5
Total	176	36

**Table 2:** Demographic characteristics of the study participants (*n* = 176) from Kajiado Central Subcounty.

Characteristics	Frequency (%) based on origin of wards					
	M. South	M. North	Purko	Ilidamati	Dalalekutuk	Total
Gender						
Male	31 (57.4)	31 (60.8)	18 (72.0)	13 (43.3)	15 (93.8)	108 (61.4)
Female	23 (42.6)	20 (39.2)	7 (28.0)	17 (56.7)	1 (6.3)	68 (38.6)
Age						
<20 years	26 (48.1)	11 (21.6)	9 (36.0)	10 (33.3)	1 (6.3)	57 (32.4)
21-30 years	8 (14.8)	14 (27.5)	8 (32.0)	8 (26.7)	9 (56.3)	47 (26.7)
31-40 years	7 (13.0)	5 (9.8)	4 (16.0)	4 (13.3)	4 (25.0)	24 (13.6)
41-50 years	4 (7.4)	4 (7.8)	1 (4.0)	5 (16.7)	2 (12.5)	16 (9.1)
>51 years	2 (3.7)	11 (21.6)	3 (12.0)	2 (6.7)	0 (0.0)	18 (10.2)
Could not remember	7 (13.0)	6 (11.8)	0 (0.0)	1 (3.3)	0 (0.0)	14 (8.0)
Contact with livestock						
Frequent	46 (85.2)	45 (88.2)	25 (100)	18 (60.0)	11 (68.8)	145 (82.4)
Infrequent	8 (14.8)	6 (11.8)	0 (0.0)	12 (40.0)	5 (31.3)	31 (17.6)
History of hospital visitation						
Yes	19 (35.2)	15 (29.4)	6 (24.0)	3 (10.0)	4 (25.0)	47 (36.4)
No	35 (64.8)	36 (70.6)	19 (76.0)	27 (90.0)	12 (75.0)	129 (73.3)
Milk consumption						
Boiled	17 (31.5)	20 (39.2)	3 (12.0)	30 (100)	0 (0.0)	70 (39.8)
Raw	17 (31.5)	26 (51.0)	22 (88.0)	0 (0.0)	16 (100)	81 (46.0)
Not sure	20 (37.0)	5 (9.8)	0 (0.0)	0 (0.0)	0 (0.0)	25 (14.2)
Antibiotic use						
Yes	9 (5.6)	3 (5.8)	1 (4.0)	3 (10.0)	0 (100)	16 (9.1)
No	45 (83.3)	48 (94.1)	24 (96.0)	27 (90.0)	16 (100)	160 (90.9)

M: Matapato.

**Table 3:** Prevalence of bacteria isolated from nasal cavities of 176 nasal swabs of participants in Kajiado Central Subcounty, Kenya.

Type	Total isolates (*N*)	Prevalence (%)
CoNS	79	44.9
*Enterococci* spp.	76	43.2
*Bacillus* spp.	14	8
*Staphylococcus aureus*	14	8
*Corynebacterium* spp.	5	2.8
*Morganella* spp.	2	1.1
*Micrococcus* spp.	1	0.6
*Lactococcus* spp.	1	0.6
*Proteus mirabilis*	1	0.6
*Pantoea* spp.	1	0.6
*Aeromonas hydrophila*	1	0.6

CoNS = coagulase-negative Staphylococcus.

**Table 4:** Antibiotic sensitivity profile of Staphylococcus isolates from nasal swabs of participants in Kajiado Central Subcounty, Kenya.

Resistance/sensitivity levels for Staphylococcus isolates							
Antibiotic	Isolate	Resistant (*n*, %)		Intermediate (*n*, %)		Susceptible (*n*, %)	
Oxacillin	CoNS	37	49.3	0	0	38	50.7
	*S. aureus*	4	28.6	1	7.14	9	64.3

Augmentin	CoNS	9	12.0	0	0	66	88.0
	*S. aureus*	0	0.0	0	0.0	14	100.0

Ceftazidime	CoNS	57	76.0	14	18.7	4	5.3
	*S. aureus*	14	100.0	0	0.0	0	0.0

Amoxicillin	CoNS	59	78.7	5	6.7	11	14.7
	*S. aureus*	10	71.4	2	14.3	2	14.3

Erythromycin	CoNS	28	37.3	14	18.7	33	44.7
	*S. aureus*	3	21.4	3	21.4	8	57.1

Tetracycline	CoNS	26	34.7	16	21.3	33	44.7
	*S. aureus*	2	14.3	2	14.3	10	71.4

Cefoxitin	CoNS	21	28.0	0	0	54	72.0
	*S. aureus*	2	14.2	0	0	11	78.6

Streptomycin	CoNS	17	22.7	21	28.0	37	49.3
	*S. aureus*	10	71.4	4	28.6	0	0.0

Vancomycin	CoNS	19	25.3	0	0	56	74.7
	*S. aureus*	5	35.7	0	0	9	64.3

Clindamycin	CoNS	11	14.7	30	40.0	34	45.3
	*S. aureus*	3	21.4	7	50.0	4	28.5

Gentamicin	CoNS	13	17.3	13	17.3	49	65.3
	*S. aureus*	0	0.0	11	78.6	3	21.4

Cefotaxime	CoNS	5	6.7	16	21.3	54	72.0
	*S. aureus*	0	0.0	2	14.3	12	85.7

Ceftriaxone	CoNS	3	4.0	25	33.3	47	62.7
	*S. aureus*	2	14.3	1	7.1	11	78.6

Ciprofloxacin	CoNS	4	5.3	8	10.7	63	84.0
	*S. aureus*	0	0.0	3	21.4	11	78.6

**Table 5:** Antibiotic resistance combination patterns of CoNS and S. aureus isolated from nasal swabs of participants in Kajiado Central Subcounty, Kenya.

Bacteria	Antibiotic combination	No. of isolates (*n*)	%
CoNS	AMX and OX	61	77.2
	AMX and E	27	34.2
	AMX and VAN	20	25.3
	AMX and TE	24	30.4
	AMX and CTX	41	51.9
	E and CTX	23	29.1
	OX and CTX	56	70.9
	OX and VAN	20	25.3
	OX and TE	26	32.9
	OX and E	30	38.0

*S. aureus*	AMX and CTX	10	71.4
	AMX and STR	6	42.9
	CTX and STR	7	50.0
	CTX and VAN	5	35.7

Key: AMX: amoxicillin; CTX: cefotaxime; E: erythromycin; OX: oxacillin; STR: streptomycin; TE: tetracycline; VAN: vancomycin.

**Table 6:** Distribution of *Staphylococcus* spp. antibiotic resistance in participants from Kajiado Central Subcounty, Kenya.

	Wards					Total (*n*, %)
	M. South (*n*, %)	M. North (*n*, %)	Ilidamati (*n*, %)	Purko (*n*, %)	Dalalekutuk (*n*, %)	
Beta-lactams	44 (100)	31 (100)	7 (100)	12 (92.3)	4 (100)	98 (99.0)
Macrolides	19 (43.2)	15 (48.4)	2 (28.6)	1 (7.7)	0 (0.0)	37 (37.7)
Glycopeptide	15 (34.1)	6 (19.4)	1 (14.3)	0 (0.0)	0 (0.0)	22 (22.2)
Aminoglycosides	11 (25.0)	6 (19.4)	1 (14.3)	4 (30.8)	1 (25.0)	23 (23.2)
Tetracycline	10 (22.7)	8 (25.8)	3 (42.9)	6 (46.2)	0 (0.0)	27 (23.3)
Quinolones	2 (4.5)	0 (0.0)	0 (0)	0 (0.0)	0 (0.0)	2 (2.0)

Key: M = Matapato.

**Table 7:** Relationship between various factors and the occurrence of MDR bacteria in participants from Kajiado Central Subcounty, Kenya.

Factor	Number	MDR prevalence	*p* value, odds ratio
Gender	Male	98.1	*p* = 0.02, OR = 0.63
	Female	100	

Age	<40	96.4	*p* > 0.05, OR = 1.61
	>40	100	

Intake of raw milk	Yes	100	*p* = 0.02, OR = 1.82
	No	98.3	

Animal contact	Frequently	98.8	*p* = 1, OR = 0.94
	Infrequently	100	

Hospital visitation (≤3 months)	Yes	15.6	*p* = 0.5, OR = 0.72
	No	84.4	

Antibiotic usage (≤3 months)	Yes	15.6	*p* = 0.07, OR = 1.0
	No	84.4	

## Data Availability

The datasets generated are available from the corresponding author on reasonable request.
